# Genomics of Peripheral T-Cell Lymphoma and Its Implications for Personalized Medicine

**DOI:** 10.3389/fonc.2020.00898

**Published:** 2020-06-19

**Authors:** Yumeng Zhang, Dasom Lee, Thomas Brimer, Mohammad Hussaini, Lubomir Sokol

**Affiliations:** ^1^Department of Internal Medicine, University of South Florida, Tampa, FL, United States; ^2^Department of Hematopathology, H. Lee Moffitt Cancer Center and Research Institute, Tampa, FL, United States; ^3^Department of Malignant Hematology, H. Lee Moffitt Cancer Center and Research Institute, Tampa, FL, United States

**Keywords:** PTCL, AITL, ALCL, PTCL-NOS, genomics, personalized medicine, diagnosis, management

## Abstract

Peripheral T-cell lymphoma (PTCL) is a rare, heterogenous group of mature T-cell neoplasms that comprise 10–15% of non-Hodgkin lymphoma cases in the United States. All subtypes of PTCL, except for ALK^+^ anaplastic T-cell lymphoma, are associated with poor prognosis, with median overall survival (OS) rates of 1–3 years. The diagnosis of PTCL is mainly based on clinical presentation, morphologic features, and immunophenotypes. Recent advances in genome sequencing and gene expression profiling have given new insights into the pathogenesis and molecular biology of PTCL. An enhanced understanding of its genomic landscape holds the promise of refining the diagnosis, prognosis, and management of PTCL. In this review, we examine recently discovered genetic abnormalities identified by molecular profiling in 3 of the most common types of PTCL: *RHOA*^*G*17*V*^ and epigenetic regulator mutations in angioimmunoblastic T-cell lymphoma, ALK expression and JAK/STAT3 pathway mutations in anaplastic T-cell lymphoma, and T-follicular helper phenotype and GATA3/TBX21 expression in PTCL-not otherwise specified. We also discuss the implications of these abnormalities for clinical practice, new/potential targeted therapies, and the role of personalized medicine in the management of PTCL.

## Introduction

Peripheral T-cell lymphoma (PTCL) is a rare and heterogeneous group of mature T-cell neoplasms, comprising 10–15% of all cases of non-Hodgkin lymphoma cases in the United States (1), with at least 29 subtypes recognized by the revised 2016 World Health Organization classification of lymphoid neoplasms (2). PTCL generally carries a poor prognosis. The complex pathobiology of these disorders is well-reflected in their heterogeneous clinical, histological, and immunophenotypic features. Advances in the genome sequencing and gene expression profiling (GEP) of PTCL have improved our understanding of its molecular pathobiology, and a precise definition of its molecular background has revealed novel therapeutic targets. In this review, we focus on the recently discovered somatic genetic abnormalities of and emerging therapies for 3 of the most common PTCL subtypes: angioimmunoblastic T-cell lymphoma (AITL), anaplastic large cell lymphoma (ALCL), and PTCL-not otherwise specified (PTCL-NOS) (Supplementary Table 1). Germline mutations are beyond the scope of this review.

## Angioimmunoblastic T-Cell Lymphoma and Other Nodal Lymphomas of Follicular T Helper Cell Origin

AITL is the one of the most common PTCL entities, accounting for ~20–30% of all PTCL cases in the United States (3). The AITL cell originates from CD4^+^ T follicular helper cells (TFHs) (4). AITL is associated with B-cell lymphoproliferative disorders and a constitutively activated immune system (5). The prognosis of patients with AITL is poor, with a 5-year overall survival (OS) rate of 33% (5). There has been no significant improvement in OS in the last 3 decades (6).

Recent advances in next-generation sequencing have led to the discovery of recurrent mutations in AITL. The most frequently reported somatic mutations include alterations in epigenetic regulators; ras homolog family member A (*RHOA*); and T-cell receptor (TCR) signaling pathway molecules (Table 1).

**Table 1:** Genetic aberrations reported in the 3 most common types of PTCL.

**Gene**	**Frequency, %**	**Potential Targets**	**Examples**	**Reference**
**ANGIOIMMUNOBLASTIC T-CELL LYMPHOMA**				
**RAS super family**				
*RHOA^*G*17*V*^*	50–72	Multikinase inhibitors; PI3K inhibitors	dasatinib; duvelisib	(7–10)
**Epigenetic regulators**				
*TET2*	47–86	HMAs, HDACis	5-azacytidine;romidepsin^*^	(7, 9, 11, 12)
*DNMT3A*	20–48	HMAs, HDACis	5-azacytidine; romidepsin^*^	(7, 9–11, 13)
*IDH2^*R172*^*	20–45	HMAs, HDACis	5-azacytidine; romidepsin^*^	(7, 9, 11, 14)
**TCR signaling pathway**				
*PLCγ*	14	Calcineurin inhibitors	cyclosporine A	(13)
*CD28^#^*	9–11	Calcineurin inhibitors	cyclosporine A	(15, 16)
*FYN*	3–4	Calcineurin inhibitors	cyclosporine A	(10, 15)
*VAV1*	5	RAC1 inhibitor	azathioprine	(15)
**Structural alteration**				
*CTLA4-CD28* fusion	58	Anti-CTLA4 immunotherapy	ipilimumab	(17)
*ICOS-CD28* fusion	5			(16)
**ANAPLASTIC lARGE CELL LYMPHOMA**				
**Transcription factor**				
*STAT3*	3	JAK/STAT inhibitors	ruxolitinib	(18)
*JAK1*	8	JAK/STAT inhibitors	ruxolitinib	(18)
*JAK1+STAT3*	7	JAK/STAT inhibitors	ruxolitinib	(18)
**Epigenetic regulators**				
*TET2*	33	HMAs, HDACis	5-azacytidine, romidepsin^*^	(19)
*DNMT3A*	17	HMAs, HDACis	5-azacytidine, romidepsin^*^	
**TCR signal pathway**				
*VAV1*	11	RAC1 inhibitors	azathioprine	(20)
**Tumor suppressor**				
*DUSP22*	30			(21, 22)
*TP53^#^*	42			(21, 22)
*PRDM1^#^*	35			(21, 22)
**Structural alteration**				
*ROS1*	N/A	JAK/STAT3 inhibitors	ruxolitinib	(18)
*TYK2*	N/A	JAK/STAT3 inhibitors	ruxolitinib	(18)
*ERBB4*	24	ERB Kinase Inhibitors	cetuximab, gefitinib	(23)
*COL29A1*	24	ERB Kinase Inhibitors	cetuximab, gefitinib	(23)
*TP63* rearrangement^#^	8			(21)
**PERIPHERAL T-CELL LYMPHOMA, NOT OTHERWISE SPECIFIED**				
**Epigenetic regulator**				
*TET2*	38–49	HMAs, HDACis	romidepsin*, belinostat*, azacytidine	(9, 12, 24–26)
*DNMT3A*	5–27	HMAs, HDACis	romidepsin*, belinostat*, azacytidine	(9, 12, 24–26)
*IDH2*	0–8	HMAs, HDACis	romidepsin*, belinostat*, azacytidine	(9, 12, 24–26)
*KMT2C KMT2D*	8 2–20	HMAs, HDACis	romidepsin*, belinostat*, azacytidine	(9, 12, 24–26)
*SETD1B SETD2 KDM6A CREBBP*	5 3–10 1–11 4–16	HMAs, HDACis	romidepsin*, belinostat*, azacytidine	(9, 12, 24–27)
**Tumor suppressor**				
*TP53^#^*	7–16			(25, 28, 29)
*ATM*	4–16			
**TCR signaling pathway**				
*RHOA^G17V^*	7–26	Multikinase inhibitors; PI3K inhibitors	duvelisib, tenalisib	(9, 10, 24–26)
*FYN*	2–3	SYK inhibitors	fostamatinib, entospletinib	(9, 10, 24–26)
**Structural alteration**				
*VAV1* fusion	18	RAC1 inhibitor	azathioprine	(17, 20, 30, 31)
*CTLA4-CD28* fusion	23	Anti-CTLA4 immunotherapy	ipilimumab	(17, 20, 30, 31)
*ITK-SYK* fusion	17–18	SYK inhibitors	fostamatinib, entospletinib	(17, 20, 30, 31)

* denotes FDA approved therapy for PTCL;

# *denotes poor prognostic indicators*.

*HDACis, histone deacetylase inhibitors; HMAs, Hypomethylating agents; PI3K, phosphoinositide 3-kinase; SYK, spleen tyrosine kinase*.

### Epigenetic Regulators

Tet methylcytosine dioxygenase 2 (*TET2*), DNA methyltransferase 3α (*DNMT3A*), and mitochondrial isocitrate dehydrogenase 2 (*IDH2*) genes participate in the regulation of DNA methylation/hydromethylation. Mutations in *TET2* and *DNMT3A* are associated with hypermethylation and dysregulated gene expression (11, 32), and the *IDH2*^*R*172^ mutant confers neo-enzymatic activity, producing oncometabolite D-2 hydroxyglutarate (D-2-HG). The accumulation of D-2-HG inhibits both histone lysine demethylase and the DNA hydroxylase in the TET family (33). Interestingly, the high co-occurrence of *TET2* and *IDH2*^*R*172^ mutations in AITL suggests a synergistic effect, by which these genes upregulate follicular T helper–associated genes and downregulate genes associated with T_H1_, T_H2_, and T_H17_ cells (7, 19).

Epigenetic modulating agents are promising targets for patients with relapsed AITL. 5-azacytidine, has induced a sustained response in selected patients with *TET2*-mutated relapsed/refractory AITL (34). A similar response is reported with romidepsin (35).

### TCR Signaling Pathway

The *RHOA*^*G*17*V*^ mutation is common in AITL. RHOA is a small GTPase that mediates T-cell migration, polarity, and thymocyte development (36). Glycine at RHOA residue 17 is critical for GTP binding. Thus, the substitution of Valine leads to a loss of GTPase activity (8). It was initially believed that the *RHOA*^*G*17*V*^ mutation played an oncogenic role by disrupting the classical RHOA signaling. However, a recently reported p.K18N mutant in AITL is associated with higher GTP binding capacity (15). This phenomenon is explained by the RHOA-VAV1 signaling pathway. VAV1, a guanine exchange factor protein, functions as an adaptor to facilitate and activate the TCR proximal signaling complex. The binding of G17V RHOA to VAV1 augments VAV1's adaptor function, resulting in an accelerated TCR signaling. An isolated VAV1 mutation has also been identified in AITL (37). Dasatinib blocked accelerated VAV1 phosphorylation and TCR signaling *in vitro* and improved the overall survival of the mice model (37).

In preclinical models, the expression of RHOA^G17V^ induced TFH cell specification, upregulated the inducible co-stimulator (ICOS), and increased phosphoinositide 3-kinase (PI3K) and mitogen-activated protein kinase signaling. PI3K inhibitors efficiently inhibited TET2-/-RHOA G17V tumor proliferation (38).

Other TCR-related mutations in AITL include *PLCG1, CD28*, and *FYN*. *CD28* is the primary costimulatory receptor in T cells and induces sustained T-cell proliferation and cytokine production. The presence of *CD28* mutations correlates with a poor prognosis (16). Cyclosporine A, a calcineurin inhibitor that blocks TCR signaling, effectively prevented the progression of AITL (39, 40). Two structural changes, *CTLA4-CD28* (17) and *ICOS-CD28* fusion genes (16), have also been described. Ipilimumab, an anti-CTLA4 immunotherapy, is a potential treatment for the *CTLA4-CD28* fusion gene.

### Multistep Tumorigenesis Model

To account for the complex genomic landscape of AITL, a multistep tumorigenesis model was proposed (41–43). The premalignant hematopoietic progenitor cells harboring mutations (e.g., *TET2* and *DNMT3A*) are predisposed to the development of blood cancer, and the acquisition of second-hit mutations (e.g., *RHOA*^*G*17*V*^ and *IDH2*^*R*172^) in a subclone of TFH cells eventually leads to AITL. This model is supported by the detection of *TET2* and *DNMT3A* mutations in tumor-free peripheral blood cells, bone marrow cells, and hematopoietic progenitors, whereas *RHOA* and *IDH* mutations are specific to malignant cells from AITL tumors (13).

### Nodal T-Cell Lymphomas With TFH Phenotype as a Newly Proposed Group of PTCL

Together with AITL, nodal PTCL with TFH phenotype and follicular T-cell lymphoma (F-PTCL) belong to a newly proposed group of PTCL called “nodal T-cell lymphomas with TFH phenotype,” described in the 2016 revised WHO classification (2, 44). This change reflects the observation that a subset of PTCLs expresses TFH-associated markers (45, 46). Interestingly, this subset shares common genetic abnormalities with AITL (9, 10, 12, 14, 24, 32). The analysis of 94 cases of AITL, 5 cases of F-PTCL, and 16 cases of nodal PTCL with TFH phenotype supported this grouping (13). These entities shared not only disease severity and prognosis, but also global and specific gene expression patterns. They had similar mutation frequencies in *TET2, RHOA, DNMT3A*, with the exception of *IDH2*^*R*172^ mutation, which was restricted to AITL.

We recommend routine screening of any PTCL-NOS for TFH markers and assigning them to this new category when at least 2 TFH markers are simultaneously detected on the neoplastic cells.

## ALCL

ALCL accounts for ~12% of PTCL cases in the United States (3). ALCL is characterized by CD30 positivity. The 2016 revision of WHO classification system for lymphoid neoplasms recognizes 4 subtypes of ALCL: ALK^+^ ALCL, ALK^−^ ALCL, primary cutaneous, and breast implant–associated ALCL (44, 47). The differentiation between ALK^+^ and ALK^−^ subtypes has formed the backbone of the current classification system.

### ALK^+^ ALCL

ALK^+^ ALCL commonly presents in young populations, generally within the first 3 decades of life and carries a significantly better prognosis (5-year OS, 70–85%) than ALK^−^ ALCL (5-year OS, 30–49%) (48). The presence of *ALK* gene rearrangements in ALK^+^ ALCL, most commonly translocation t(2;5)(p23;q35), results in the fusion of nucleophosmin (NPM1) and ALK (49). Anti-ALK antibodies can identify the proteins produced by NPM1/ALK transcripts based on staining patterns. ALK^+^ ALCL expressed ALK in nucleus and cytoplasm; conversely, variant fusions lacked nuclear ALK-staining (50).

*ALK* gene rearrangements often occur within the intron, between exons 19 and 20, allowing the intracytoplasmic domain of ALK to fuse with NPM1. The dimerization domain auto-phosphorylates the ALK catalytic domain and activates multiple downstream signaling pathways, including PI3K/AKT, RAS/ERK, and JAK/STAT (51).

NPM1-ALK cell lines express STAT3 phosphorylated on serine residue 727 and tyrosine residue 705 and increase STAT3 expression at the transcriptional level. Although JAK3 is phosphorylated, its binding is not essential for STAT3 activity. NPM-ALK fusion transcripts could activate STAT3 directly (52). This activation is important, as STAT3 is integral to cell survival by controlling the transcription of numerous apoptosis-regulating proteins, such as cyclin D1, Bcl-X, Bcl-XL, and c-Myc (53). Although NPM1/ALK fusion transcripts are the most common rearrangements in ALK+ ALCL, other rearrangements, such as TPM3 (1q25), ATIC (2q35), TFG (3q21), TPM4 (19p13.1), MYH9 (22q11.2), RNF213 (17q25), TRAF1 (9q33.2), CLTC (17q23), and MSN (Xq11), have also been reported (54).

### ALK^−^ ALCL

ALK– ALCL was upgraded from a provisional to a definite entity in the revised 2016 WHO classification (55). It is difficult to differentiate between ALK– ALCL and PTCL-NOS based on CD30 positivity (20). To better define ALCL from PTCL-NOS, GEP of PTCL-NOS, and ALCL discovered a unique cluster of gene transcripts shared by ALK– and ALK+ALCLs (56). We can also distinguish ALK–ALCL from CD30+ PTCL-NOS through clinical outcomes (57, 58). CD30+ PTCL-NOS has a poorer prognosis and requires more aggressive treatment (59, 60). Based on GEP, a 3-gene model (TNFRSF8, BATF3, and TMOD1) was developed to separate ALK– ALCL from PTCL-NOS, with 97% accuracy (61).

#### Chromosomal Rearrangements of DUSP22 and TP63 as a Differentiating Factor

Two chromosomal rearrangements, *DUSP22* and *TP63*, subclassify ALK^−^ALCL into 3 groups: DUSP22-rearranged, TP63-rearranged, and group without any rearrangement. *DUSP22* rearrangement occurs in 30% of ALK^−^ ALCL patients and is associated with a 5-year OS rate of 80–90%, similar to that of ALK^+^ ALCL (5-year OS, 85%) (62). It is associated with downregulation of *DUSP22*, which leads to the inhibition of TCR signaling and the promotion of apoptosis (21). Its unique immunogenic molecular signature, such as DNA hypomethylation, lower expression of PD-1, and higher expression of costimulatory gene *CD58* and HLA Class II likely contributes to its favorable prognosis (63). Other clinical predictors, such as IPI risk factors, age, and CD3 positivity, also impact prognosis despite *DUSP22* rearrangement (64, 65).

*TP63* rearrangement, the fusion transcript of TBL1XR1/TP63, has similar structural homology to oncogenic deltaNp63 in p53 tumor suppressor pathway and is associated with inferior survival (66).

A third category, defined as triple-negative (ALK^−^, *DUSP22*^−^, and *TP63*^−^), harbors the remaining 62% of ALK^−^ ALCL cases and has a 5-year OS rate of 42% (67). Although further validation of this model is needed, *DUSP22* and *TP63* rearrangements may serve as useful biomarkers in prognosis and direct therapy for patients with ALK^−^ ALCL in the future.

#### Other Genetic Aberrations in ALK^−^ALCL

As in ALK^+^ ALCL, the JAK/STAT3 pathway is constitutively activated in ALK^−^ ALCL (68). Recurrent single or convergent somatic mutations and translocations in the *JAK1* and *STAT3* genes are thought to upregulate the STAT3 pathway (18, 19). In addition, the gene fusions involving *ROS1* and *TYK2* in some ALK^−^ ALCLs have led to the activation of STAT3 independent of JAK1 or STAT3 mutations. RNA sequencing has identified the co-expression of truncated *ERBB4* and *COL29A1* in 24% of patients with ALK^−^ ALCL (23). These *ERBB4*-truncated forms are potentially oncogenic, and *ERBB4* inhibition can partially arrest cell growth and stop disease progression. These transcripts were not observed in ALK^+^ ALCL or PTCL-NOS patients. More recently, losses at 6p21 and 17p13 were identified in ALK^−^ ALCL using single nucleotide polymorphism arrays (22, 69). These losses correlated with the losses of *TP53* and *PRDM1* and poor prognoses.

## PTCL-NOS

PTCL-NOS is the most common subtype of PTCL, accounting for 30–50% of PTCL cases in the United States (3, 70). Patients are diagnosed with PTCL-NOS if they do not meet the diagnostic criteria of other PTCL subtypes as per WHO 2016 revision (2, 70). As a diagnosis of exclusion, PTCL-NOS comprises a heterogeneous group of diseases with diverse cells of origin and presents with different cytogenic, molecular, and morphological phenotypes. This heterogeneity makes classification and treatment of the disease difficult. With the standard anthracycline-based chemotherapy, complete response rates range from 40 to 60%, and 5-year OS rates range from 30 to 40% (71, 72).

### GATA3 and TBX21 Expression as a Differentiating Factor in PTCL-NOS

PTCL-NOS can be categorized based on *GATA3* and *TBX21* expression *and* T helper 1 and T helper 2 cell differentiation regulators (73, 74). PTCL-NOS cases with high expression of *TBX21* have a tumor microenvironment gene signature, whereas those of *GATA3* have a cytotoxic gene signature with poorer outcomes (75). The greater genomic complexity associated with *GATA3* is characterized by frequent loss of tumor suppressor genes on the CDKN2A/B-TP3 axis and PTEN-PI3K pathways as well as genetic gains and amplification of *STAT3* and *MYC*. Immunohistochemistry (IHC) algorithm can be used to identified the two subtypes and add in risk stratification for clinical trials (76).

Watatani's group studied the relation between PTCL-NOS with TFH phenotype and *GATA3/TBX21* expression using GEP (25). PTCL-NOS without TFH phenotype often has mutations in *TP53* and/or *CDKNA2A* genes, which can cause chromosomal instability and mediate immune escape and transcriptional regulations. Those mutations potentially explain the worse outcomes among patients with PTCL-NOS without TFH phenotype as compared with those with TFH phenotype. However, there was no difference in *GATA3* and *TBX21* expression in the TFH-related group and in the *TP53/CDKN2A*-altered group.

### Other Genetic Aberrations in PTCL-NOS

The *FYN* gene encodes a tyrosinase kinase involved in T-cell activation and Src kinase inhibition. Dasatinib targeted the Src kinase *in vitro*, and could be a l target for patients with mutations in *FYN* genes (10). Recurrent loss at 9p21.3 decreases the expression of the cyclin-dependent kinase inhibitors 2A and 2B which are associated with a poorer prognosis (28). Guanine nucleotide exchange factor *VAV1* encodes a critical component of TCR signaling, and recurrent gene fusion of *VAV1* has also been identified (20). Recurrent genetic activating mutations and translocations of *VAV1* gene in PTCL-NOS highlighted its role of a drive oncogene in catalytic-dependent (MAPK and JNK) and -independent (NFAT) VAV1 effector pathways (77). Azathioprine targets cells overexpressing the *VAV1-GSS* fusion protein (20). *CTLA4-CD28* fusion and mutations in *KMT2C* and *SET1B* (histone methylation) have also been identified (17, 27).

*FAT1* tumor suppressor binds to β-catenin and inhibits nuclear localization, thus inhibiting cell growth. The recurrent mutations in *FAT1* tumor suppressor gene were seen in 39% cases of PTCL-NOS and is associated with inferior outcome (29).

With new differentiating factors such as TFH phenotype, *GATA3/TBX21* expression, we may expect PTCL-NOS to be better categorized, which could provide more insight into defining targetable molecular pathways and developing novel therapeutic strategies for PTCL-NOS patients.

## Clinical Implications and Personalized Medicine

Recent advances in genome sequencing and GEP have led to the identification of commonly dysregulated pathways, especially enhanced T-cell signaling pathways, in all of the 3 most common nodal subtypes of PTCL. Despite similarities in genomic profiles, the interaction between various pathways might play a role in determining divergent cell differentiation and tumorigenesis. For example, some mutations in the epigenetic modifier genes are similar between AITL and myeloid neoplasms; however, the mutation patterns are different. *TET2* and *IDH2* mutations are mutually exclusive in acute myeloid leukemia. In contrast, *IDH2* mutations often cooccur with *TET2* mutations in AITL (19, 78). These different mutation patterns suggest that the interaction between *IDH2* and *TET2* mutations possibly lead to the development of a TFH phenotype.

**Figure 1:**
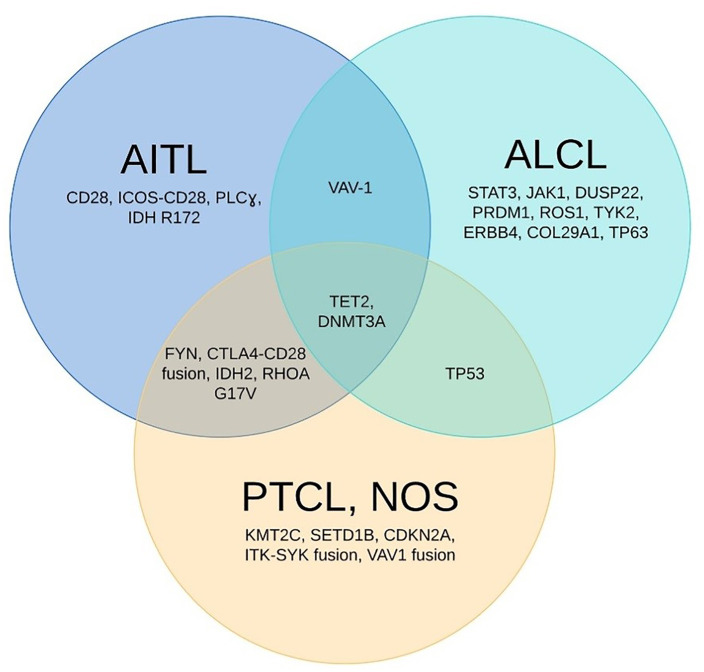
Unique and shared mutations identified in the 3 most common types of PTCL. The Venn diagram above showed the most frequently encountered genomic abnormalities in 3 most common types of peripheral T-cell lymphoma: angioimmunoblastic T-cell lymphoma (AITL), anaplastic large cell lymphoma (ALCL), and peripheral T-cell lymphoma-not otherwise specified (PTCL-NOS). TET2 and DNMT3A mutations are seen in all 3 major subtypes.

The different patterns of molecular signature profiles can help us to identify and to reclassify AITL and ALCL from PTCL-NOS in cases that do not meet morphological criteria (Figure 1). Iqbal et al. reclassified 14% of PTCL-NOS cases as AITL via GEP using the 3 prominent AITL signatures: B-cell signature, follicular dendritic-cell signature, and cytokine signature (79). This reclassification was then confirmed by the presence of the *IDH2*^R172^ mutation. The presence of the *RHOA*^G17V^ mutation helped to identify nodal T-cell lymphomas with TFH phenotype, as this mutation key in TFH cell speciation and AITL transformation (38). *ITK-SYK* gene fusion could potentially differentiate a subset of PTCL-NOS patients with TFH phenotype from those with AITL (30, 31, 80, 81) (Figure 1).

Agnelli et al. reclassified 11% of PTCL-NOS cases as ALCL using a 3-gene model (*TNFRSF8, BATF3*, and *TMOD1*) with 97% accuracy (61). Similarly, lack of *ERBB4* transcript in other PTCL subtypes could help to confirm the diagnosis of ALK^−^ ALCL (23) (Figure 1).

Furthermore, many mutations have been associated with the prognosis of PTCL. The mutations associated with a poor prognosis include *CD28* mutations in AITL (16); *TP63* rearrangement (21), loss of *TP53*, and loss of *PRDM1* (22) in ALK^−^ ALCL; *GATA3* (75), *TP53*, and/or *CDKN2A* (25) in ALK^−^ ALCL; and alterations in histone methyltransferase genes *KMT2A, KMT2B*, or *KDM6A* (82) and *FAT1* (29) in PTCL-NOS. The mutations associated with a favorable prognosis include the presence of *DUSP22* in ALK^−^ ALCL (21).

## Potential Therapeutic Targets

Most PTCL subgroups have median OS rates of 1–3 years, except for ALK^+^ ALCL (3). Only a small portion (20%-40%) of patients with PTCL achieve long-term survival (6). During the past 3 decades, long-term survival has not been significantly improved by available therapies (83). Fortunately, a greater understanding of the pathogenesis of the major PTCL subtypes has led to the identification of potentially actionable biologic pathways, especially activating pathways. Novel targeting therapies are now available for clinical studies (Table 1).

Epigenetic modulators, such as romidepsin (NCT03141203, NCT00426764), chidamide (NCT03268889), and HBI-8000 (NCT02953652), are currently being investigated in clinical trials for PTCL, either as a monotherapy or in conjunction with other therapies.

The T-cell signaling pathway also has multiple candidate therapies: multikinase inhibitors, such as dasatinib (NCT01609816, NCT01643603), for PTCL; PI3K inhibitors, such as duvelisib (NCT03372057) and copanlisib (NCT03052933), for PTCL; an anti-ICOS monoclonal antibody in ICOS-PI3K pathways, MEDI−570 (NCT02520791), for the follicular variant of PTCL-NOS and AITL; and a CTLA-4 inhibitor, such as ipilimumab, for CLA-CD28 fusion-positive tumors.

The transcription factor NF-κB pathway is differentially activated in PTCL, especially in the AITL subtype (79). Bortezomib, a proteasome inhibitor with NF-κB inhibitory activity, has shown early promise in the treatment of adult T-cell lymphoma (84). An additional clinical study on bortezomib is currently underway (NCT04061772), as well as studies on ixazomib, a drug similar to bortezomib (NCT03547700).

The JAK-STAT3 pathways are sometimes aberrantly activated in PTCL, especially in ALCL and in some cases of PTCL-NOS. JAK inhibitors, such as ruxolitinib, were previously used extensively for myeloid disorder and are currently under investigation for treating patients with PTCL (NCT01431209).

Another promising target is SYK, a receptor-associated tyrosine kinase expressed in 94% of all PTCL patients (85). The SYK inhibitor R406 effectively caused apoptosis and inhibited cell growth in preclinical studies (86). SYK inhibitors, such as entospletinib, are promising potential agents.

The ALK-1 inhibitor crizotinib was used in patients with relapsed pediatric ALK^+^ ALCL, with a complete response rate of 83% (NCT00939770) (87). For ALK^+^ ALCL resistant to crizotinib, platelet-derived growth factor receptor-β (PDGFRB) blockade is potentially effective. Imatinib, acting on PDGFRA and PDGFRB blockade, induced a complete remission in a late stage NPM-ALK^+^ ALCL patient. As suggested in murine model, ALK promotes the expression of activator protein 1 family members JUN and JUNB, which subsequently promote tumor dissemination through PDGFRB regulation (88).

Other targeted agents tested in PTCL include BCL2 inhibitors (e.g., venetoclax [NCT03552692]); monoclonal antibodies targeting CD2 (e.g., siplizumab [NCT01445535]); CCR4 monoclonal antibodies (e.g., mogalizumab [NCT01611142]); *FYN* inhibitors; CD30 antibody drug conjugates (e.g., brentuximab [NCT01716806, NCT03496779]); ERB kinase inhibitors; VGEFR-2 inhibitors (e.g., apatinib [NCT03631862]); and PD-1 and PD-L1 inhibitors for immune modulation (e.g., pembrolizumab [NCT02535247], nivolumab [NCT03586999], durvalumab [NCT03161223], and avelumab [NCT03046953]).

Currently, novel therapies are being developed rapidly, and personalized medicine is made possible through commercial gene sequencing. The genetic heterogeneity in PTCL requires an individualized therapeutic approach that uses agents that specifically target genetic abnormalities or oncogenic pathways found in patients' tumors. More rationally designed clinical trials enrolling patients with specific genetic alterations are needed to provide higher response rates and more sustained responses. In the context of genome sequencing and GEP, targeted and personalized therapies will likely provide the best clinical outcomes in patients with PTCL in the near future.

## Author Contributions

LS and MH conceptualized the idea for the manuscript and critically reviewed and edited the manuscript. YZ, DL, TB reviewed the relevant literature and collected the data, and wrote the original draft. YZ prepared the table and graph.

## Conflict of Interest

LS and MH are associate guest editors of Frontiers in Oncology; during the course of writing and submitting this paper to the journal they were recused from the editorial decision-making process, and another associate editor handled the peer-review procedures independently. The remaining authors declare that the research was conducted in the absence of any commercial or financial relationships that could be construed as a potential conflict of interest.

## References

[1] Armitage JO. The aggressive peripheral T-cell lymphomas: 2012 update on diagnosis, risk stratification, and management. Am Hematol J. (2012) 87:511–9. 10.1002/ajh.23144 22508369

[2] Swerdlow SH Campo E Pileri SA Harris NL Stein H Siebert R. The 2016 revision of the World Health Organization classification of lymphoid neoplasms. Blood. (2016) 127:2375–90. 10.1182/blood-2016-01-643569 26980727 PMC4874220

[3] Vose J Armitage J Weisenburger D International T-Cell Lymphoma Project. International peripheral T-cell and natural killer/T-cell lymphoma study: pathology findings and clinical outcomes. J Clin Oncol. (2008) 26:4124–30. 10.1200/JCO.2008.16.4558 18626005

[4] de Leval L Rickman DS Thielen C Reynies A Huang YL Delsol G. The gene expression profile of nodal peripheral T-cell lymphoma demonstrates a molecular link between angioimmunoblastic T-cell lymphoma. (AITL) and follicular helper T. (TFH) cells. Blood. (2007) 109:4952–63. 10.1182/blood-2006-10-055145 17284527

[5] Federico M Rudiger T Bellei M Nathwani BN Luminari S Coiffier B. Clinicopathologic characteristics of angioimmunoblastic T-cell lymphoma: analysis of the international peripheral T-cell lymphoma project. J Clin Oncol. (2013) 31:240–6. 10.1200/JCO.2011.37.3647 22869878 PMC3532394

[6] Crozier JA Sher T Yang D Swaika A Foran J Ghosh R. Persistent disparities among patients with T-cell non-hodgkin lymphomas and B-cell diffuse large cell lymphomas over 40 years: a SEER database review. Clin Lymphoma Myeloma Leuk. (2015) 15:578–85. 10.1016/j.clml.2015.06.005 26198444 PMC7546202

[7] Steinhilber J Mederake M Bonzheim I Serinsoz-Linke E Muller I Fallier-Becker P. The pathological features of angioimmunoblastic T-cell lymphomas with IDH2(R172) mutations. Mod Pathol. (2019) 32:1123–34. 10.1038/s41379-019-0254-4 30952970

[8] Yoo HY Sung MK Lee SH Kim S Lee H Park S. A recurrent inactivating mutation in RHOA GTPase in angioimmunoblastic T cell lymphoma. Nat Genet. (2014) 46:371–5. 10.1038/ng.2916 24584070

[9] Sakata-Yanagimoto M Enami T Yoshida K Shiraishi Y Ishii R Miyake Y. Somatic RHOA mutation in angioimmunoblastic T cell lymphoma. Nat Genet. (2014) 46:171–5. 10.1038/ng.2872 24413737

[10] Palomero T Couronne L Khiabanian H Kim MY Ambesi-Impiombato A Perez-Garcia A. Recurrent mutations in epigenetic regulators, RHOA and FYN kinase in peripheral T cell lymphomas. Nat Genet. (2014) 46:166–70. 10.1038/ng.2873 24413734 PMC3963408

[11] Odejide O Weigert O Lane AA Toscano D Lunning MA Kopp N. A targeted mutational landscape of angioimmunoblastic T-cell lymphoma. Blood. (2014) 123:1293–6. 10.1182/blood-2013-10-531509 24345752 PMC4260974

[12] Lemonnier F Couronne L Parrens M Jais JP Travert M Lamant L. Recurrent TET2 mutations in peripheral T-cell lymphomas correlate with TFH-like features and adverse clinical parameters. Blood. (2012) 120:1466–9. 10.1182/blood-2012-02-408542 22760778

[13] Dobay MP Lemonnier F Missiaglia E Bastard C Vallois D Jais JP. Integrative clinicopathological and molecular analyses of angioimmunoblastic T-cell lymphoma and other nodal lymphomas of follicular helper T-cell origin. Haematologica. (2017) 102:e148–51. 10.3324/haematol.2016.158428 28082343 PMC5395128

[14] Cairns RA Iqbal J Lemonnier F Kucuk C de Leval L Jais JP. IDH2 mutations are frequent in angioimmunoblastic T-cell lymphoma. Blood. (2012) 119:1901–3. 10.1182/blood-2011-11-391748 22215888 PMC3293643

[15] Vallois D Dobay MP Morin RD Lemonnier F Missiaglia E Juilland M. Activating mutations in genes related to TCR signaling in angioimmunoblastic and other follicular helper T-cell-derived lymphomas. Blood. (2016) 128:1490–502. 10.1182/blood-2016-02-698977 27369867

[16] Rohr J Guo S Huo J Bouska A Lachel C Li Y. Recurrent activating mutations of CD28 in peripheral T-cell lymphomas. Leukemia. (2016) 30:1062–70. 10.1038/leu.2015.357 26719098 PMC5688878

[17] Yoo HY Kim P Kim WS Lee SH Kim S Kang SY. Frequent CTLA4-CD28 gene fusion in diverse types of T-cell lymphoma. Haematologica. (2016) 101:757–63. 10.3324/haematol.2015.139253 26819049 PMC5013939

[18] Crescenzo R Abate F Lasorsa E Tabbo F Gaudiano M Chiesa N. Convergent mutations and kinase fusions lead to oncogenic STAT3 activation in anaplastic large cell lymphoma. Cancer Cell. (2015) 27:516–32. 10.1016/j.ccell.2015.03.006 25873174 PMC5898430

[19] Wang C McKeithan TW Gong Q Zhang W Bouska A Rosenwald A. IDH2R172 mutations define a unique subgroup of patients with angioimmunoblastic T-cell lymphoma. Blood. (2015) 126:1741–52. 10.1182/blood.V124.21.3580.3580 26268241 PMC4600014

[20] Boddicker RL Razidlo GL Dasari S Zeng Y Hu G Knudson RA. Integrated mate-pair and RNA sequencing identifies novel, targetable gene fusions in peripheral T-cell lymphoma. Blood. (2016) 128:1234–45. 10.1182/blood-2016-03-707141 27297792 PMC5009513

[21] Parrilla Castellar ER Jaffe ES Said JW Swerdlow SH Ketterling RP Knudson RA. ALK-negative anaplastic large cell lymphoma is a genetically heterogeneous disease with widely disparate clinical outcomes. Blood. (2014) 124:1473–80. 10.1182/blood-2014-04-571091 24894770 PMC4148769

[22] Boi M Rinaldi A Kwee I Bonetti P Todaro M Tabbo F. PRDM1/BLIMP1 is commonly inactivated in anaplastic large T-cell lymphoma. Blood. (2013) 122:2683–93. 10.1182/blood-2013-04-497933 24004669

[23] Scarfo I Pellegrino E Mereu E Kwee I Agnelli L Bergaggio E. Identification of a new subclass of ALK-negative ALCL expressing aberrant levels of ERBB4 transcripts. Blood. (2016) 127:221–32. 10.1182/blood-2014-12-614503 26463425

[24] Manso R Gonzalez-Rincon J Rodriguez-Justo M Roncador G Gomez S Sanchez-Beato M. Overlap at the molecular and immunohistochemical levels between angioimmunoblastic T-cell lymphoma and a subgroup of peripheral T-cell lymphomas without specific morphological features. Oncotarget. (2018) 9:16124–33. 10.18632/oncotarget.24592 29662631 PMC5882322

[25] Watatani Y Sato Y Miyoshi H Sakamoto K Nishida K Gion Y. Molecular heterogeneity in peripheral T-cell lymphoma, not otherwise specified revealed by comprehensive genetic profiling. Leukemia. (2019) 33:2867–83. 10.1038/s41375-019-0473-1 31092896

[26] Laribi K Alani M Truong C Baugier de Materre A. Recent advances in the treatment of peripheral T-cell lymphoma. Oncologist. (2018) 23:1039–53. 10.1634/theoncologist.2017-0524 29674443 PMC6192612

[27] Ji MM Huang YH Huang JY Wang ZF Fu D Liu H. Histone modifier gene mutations in peripheral T-cell lymphoma not otherwise specified. Haematologica. (2018) 103:679–87. 10.3324/haematol.2017.182444 29305415 PMC5865443

[28] Fujiwara SI Yamashita Y Nakamura N Choi YL Ueno T Watanabe H. High-resolution analysis of chromosome copy number alterations in angioimmunoblastic T-cell lymphoma and peripheral T-cell lymphoma, unspecified, with single nucleotide polymorphism-typing microarrays. Leukemia. (2008) 22:1891–8. 10.1038/leu.2008.191 18633432

[29] Laginestra MA Cascione L Motta G Fuligni F Agostinelli C Rossi M. Whole exome sequencing reveals mutations in FAT1 tumor suppressor gene clinically impacting on peripheral T-cell lymphoma not otherwise specified. Mod Pathol. (2020) 33:179–87. 10.1038/s41379-019-0279-8 31028364 PMC6994417

[30] Huang Y Moreau A Dupuis J Streubel B Petit B Le Gouill S. Peripheral T-cell lymphomas with a follicular growth pattern are derived from follicular helper T cells. (TFH) and may show overlapping features with angioimmunoblastic T-cell lymphomas. Am J Surg Pathol. (2009) 33:682–90. 10.1097/PAS.0b013e3181971591 19295409 PMC4838638

[31] Streubel B Vinatzer U Willheim M Raderer M Chott A. Novel t(5;9)(q33;q22) fuses ITK to SYK in unspecified peripheral T-cell lymphoma. Leukemia. (2006) 20:313–8. 10.1038/sj.leu.2404045 16341044

[32] Couronne L Bastard C Bernard OA. TET2 and DNMT3A mutations in human T-cell lymphoma. N Engl J Med. (2012) 366:95–6. 10.1056/NEJMc1111708 22216861

[33] Yang H Ye D Guan KL Xiong Y. IDH1 and IDH2 mutations in tumorigenesis: mechanistic insights and clinical perspectives. Clin Cancer Res. (2012) 18:5562–71. 10.1158/1078-0432.CCR-12-1773 23071358 PMC3897211

[34] Lemonnier F Dupuis J Sujobert P Tournillhac O Cheminant M Sarkozy C. Treatment with 5-azacytidine induces a sustained response in patients with angioimmunoblastic T-cell lymphoma. Blood. (2018) 132:2305–9. 10.1182/blood-2018-04-840538 30279227

[35] Pro B Horwitz SM Prince HM Foss FM Sokol L Greenwood M. Romidepsin induces durable responses in patients with relapsed or refractory angioimmunoblastic T-cell lymphoma. Hematol Oncol. (2017) 35:914–7. 10.1002/hon.2320 27402335 PMC5763404

[36] Zhang S Konstantinidis DG Yang JQ Mizukawa B Kalim K Lang RA. Gene targeting RhoA reveals its essential role in coordinating mitochondrial function and thymocyte development. Immunol J. (2014) 193:5973–82. 10.4049/jimmunol.1400839 25398325 PMC4258484

[37] Fujisawa M Sakata-Yanagimoto M Nishizawa S Komori D Gershon P Kiryu M. Activation of RHOA-VAV1 signaling in angioimmunoblastic T-cell lymphoma. Leukemia. (2018) 32:694–702. 10.1038/leu.2017.273 28832024 PMC5843900

[38] Cortes JR Ambesi-Impiombato A Couronne L Quinn SA Kim CS da Silva Almeida AC. RHOA G17V induces T follicular helper cell specification and promotes lymphomagenesis. Cancer Cell. (2018) 33:259–73.e7. 10.1016/j.ccell.2018.01.001 29398449 PMC5811310

[39] Advani R Horwitz S Zelenetz A Horning SJ. Angioimmunoblastic T cell lymphoma: treatment experience with cyclosporine. Leuk Lymphoma. (2007) 48:521–5. 10.1080/10428190601137658 17454592

[40] Chen XG Huang H Tian Y Guo CC Liang CY Gong YL Cyclosporine, prednisone, and high-dose immunoglobulin treatment of angioimmunoblastic T-cell lymphoma refractory to prior CHOP or CHOP-like regimen. Chin Cancer J. (2011) 30:731–8. 10.5732/cjc.011.10071 PMC4012273 21959050

[41] Sakata-Yanagimoto M. Multistep tumorigenesis in peripheral T cell lymphoma. Int Hematol J. (2015) 102:523–7. 10.1007/s12185-015-1738-8 25628103

[42] Lemonnier F Gaulard P de Leval L. New insights in the pathogenesis of T-cell lymphomas. Curr Opin Oncol. (2018) 30:277–84. 10.1097/CCO.0000000000000474 30028743

[43] Fukumoto K Nguyen TB Chiba S Sakata-Yanagimoto M. Review of the biologic and clinical significance of genetic mutations in angioimmunoblastic T-cell lymphoma. Cancer Sci. (2018) 109:490–6. 10.1111/cas.13393 28889481 PMC5834775

[44] IARC WHO Classification of Tumours of Haematopoietic and Lymphoid Tissues, 4th ed (2017).

[45] Agostinelli C Hartmann S Klapper W Korkolopoulou P Righi S Marafioti T. Peripheral T cell lymphomas with follicular T helper phenotype: a new basket or a distinct entity? Revising Karl Lennert's personal archive. Histopathology. (2011) 59:679–91. 10.1111/j.1365-2559.2011.03981.x 22014049

[46] Pileri SA. Follicular helper T-cell-related lymphomas. Blood. (2015) 126:1733–4. 10.1182/blood-2015-08-665075 26450950

[47] Stein H Foss HD Durkop H Marafioti T Delsol G Pulford K Pileri S. CD30(+) anaplastic large cell lymphoma: a review of its histopathologic, genetic, clinical features. Blood. (2000) 96:3681–95. 10.1182/blood.V96.12.3681 11090048

[48] Moffitt AB Dave SS. Clinical applications of the genomic landscape of aggressive non-hodgkin lymphoma. J Clin Oncol. (2017) 35:955–962. 10.1200/JCO.2016.71.7603 28297626

[49] Abate F Zairis S Ficarra E Acquaviva A Wiggins CH Frattini V. Pegasus: a comprehensive annotation and prediction tool for detection of driver gene fusions in cancer. BMC Syst Biol. (2014) 8:97. 10.1186/s12918-014-0097-z 25183062 PMC4363948

[50] Damm-Welk C Klapper W Oschlies I Gesk S Rottgers S Bradtke J. Distribution of NPM1-ALK and X-ALK fusion transcripts in paediatric anaplastic large cell lymphoma: a molecular-histological correlation. Br Haematol J. (2009) 146:306–9. 10.1111/j.1365-2141.2009.07754.x 19545284

[51] Velusamy T Kiel MJ Sahasrabuddhe AA Rolland D Dixon CA Bailey NG. A novel recurrent NPM1-TYK2 gene fusion in cutaneous CD30-positive lymphoproliferative disorders. Blood. (2014) 124:3768–71. 10.1182/blood-2014-07-588434 25349176

[52] Ducray SP Natarajan K Garland GD Turner SD Egger G. The transcriptional roles of ALK fusion proteins in tumorigenesis. Cancers. (2019) 11:E1074. 10.3390/cancers11081074 31366041 PMC6721376

[53] Zamo A Chiarle R Piva R Howes J Fan Y Chilosi M. Anaplastic lymphoma kinase. (ALK) activates Stat3 and protects hematopoietic cells from cell death. Oncogene. (2002) 21:1038–47. 10.1038/sj.onc.1205152 11850821

[54] Xing X Feldman AL. Anaplastic large cell lymphomas: ALK positive, ALK negative, primary cutaneous. Adv Anat Pathol. (2015) 22:29–49. 10.1097/PAP.0000000000000047 25461779

[55] Sandell RF Boddicker RL Feldman AL. Genetic landscape and classification of peripheral T cell lymphomas. Curr Oncol Rep. (2017) 19:28. 10.1007/s11912-017-0582-9 28303495 PMC5517131

[56] Piva R Agnelli L Pellegrino E Todoerti K Grosso V Tamagno I. Gene expression profiling uncovers molecular classifiers for the recognition of anaplastic large-cell lymphoma within peripheral T-cell neoplasms. J Clin Oncol. (2010) 28:1583–90. 10.1200/JCO.2008.20.9759 20159827

[57] Piccaluga PP Fuligni F De Leo A Bertuzzi C Rossi M Bacci F. Molecular profiling improves classification and prognostication of nodal peripheral T-cell lymphomas: results of a phase III diagnostic accuracy study. J Clin Oncol. (2013) 31:3019–25. 10.1200/JCO.2012.42.5611 23857971

[58] Savage KJ Harris NL Vose JM Ullrich F Jaffe ES Connors JM. ALK- anaplastic large-cell lymphoma is clinically and immunophenotypically different from both ALK+ ALCL and peripheral T-cell lymphoma, not otherwise specified: report from the International Peripheral T-Cell Lymphoma Project. Blood. (2008) 111:5496–504. 10.1182/blood-2008-01-134270 18385450

[59] Mereu E Pellegrino E Scarfo I Inghirami G Piva R. The heterogeneous landscape of ALK negative ALCL. Oncotarget. (2017) 8:18525–36. 10.18632/oncotarget.14503 28061468 PMC5392347

[60] Piccaluga PP Agostinelli C Califano A Rossi M Basso K Zupo S. Gene expression analysis of peripheral T cell lymphoma, unspecified, reveals distinct profiles and new potential therapeutic targets. J Clin Invest. (2007) 117:823–34. 10.1172/JCI26833 17304354 PMC1794115

[61] Agnelli L Mereu E Pellegrino E Limongi T Kwee I Bergaggio E. Identification of a 3-gene model as a powerful diagnostic tool for the recognition of ALK-negative anaplastic large-cell lymphoma. Blood. (2012) 120:1274–81. 10.1182/blood-2012-01-405555 22740451

[62] Pedersen MB Hamilton-Dutoit SJ Bendix K Ketterling RP Bedroske PP Luoma IM. DUSP22 and TP63 rearrangements predict outcome of ALK-negative anaplastic large cell lymphoma: a Danish cohort study. Blood. (2017) 130:554–7. 10.1182/blood-2016-12-755496 28522440 PMC5533203

[63] Luchtel RA Dasari S Oishi N Pedersen MB Hu G Rech KL. Molecular profiling reveals immunogenic cues in anaplastic large cell lymphomas with DUSP22 rearrangements. Blood. (2018) 132:1386–98. 10.1182/blood-2018-03-838524 30093402 PMC6161771

[64] Sibon D Nguyen DP Schmitz N Suzuki R Feldman AL Gressin R. Systemic ALK-positive anaplastic large-cell lymphoma. (ALCL): final analysis of an international, individual patient data study of 263 adults. Blood. (2017) 130:1514. 10.1182/blood.V130.Suppl_1.1514.151428774880

[65] Hapgood G Ben-Neriah S Mottok A Lee DG Robert K Villa D. Identification of high-risk DUSP22-rearranged ALK-negative anaplastic large cell lymphoma. Br Haematol J. (2019) 186:e28–31. 10.1111/bjh.15860 30873584 PMC7679007

[66] Vasmatzis G Johnson SH Knudson RA Ketterling RP Braggio E Fonseca R. Genome-wide analysis reveals recurrent structural abnormalities of TP63 and other p53-related genes in peripheral T-cell lymphomas. Blood. (2012) 120:2280–9. 10.1182/blood-2012-03-419937 22855598 PMC5070713

[67] Melard P Idrissi Y Andrique L Poglio S Prochazkova-Carlotti M Berhouet S. Molecular alterations and tumor suppressive function of the DUSP22. (Dual Specificity Phosphatase 22) gene in peripheral T-cell lymphoma subtypes. Oncotarget. (2016) 7:68734–48. 10.18632/oncotarget.11930 27626696 PMC5356586

[68] Chen J Zhang Y Petrus MN Xiao W Nicolae A Raffeld M. Cytokine receptor signaling is required for the survival of ALK- anaplastic large cell lymphoma, even in the presence of JAK1/STAT3 mutations. Proc Natl Acad Sci USA. (2017) 114:3975–80. 10.1073/pnas.1700682114 28356514 PMC5393253

[69] Feldman AL Dogan A Smith DI Law ME Ansell SM Johnson SH. Discovery of recurrent t(6;7)(p25.3;q32.3) translocations in ALK-negative anaplastic large cell lymphomas by massively parallel genomic sequencing. Blood. (2011) 117:915–9. 10.1182/blood-2010-08-303305 21030553 PMC3035081

[70] Campo E Swerdlow SH Harris NL Pileri S Stein H Jaffe ES. The 2008 WHO classification of lymphoid neoplasms and beyond: evolving concepts and practical applications. Blood. (2011) 117:5019–32. 10.1182/blood-2011-01-293050 21300984 PMC3109529

[71] Gallamini A Stelitano C Calvi R Bellei M Mattei D Vitolo U. Peripheral T-cell lymphoma unspecified. (PTCL-U): a new prognostic model from a retrospective multicentric clinical study. Blood. (2004) 103:2474–9. 10.1182/blood-2003-09-3080 14645001

[72] Weisenburger DD Savage KJ Harris NL Gascoyne RD Jaffe ES MacLennan KA. Peripheral T-cell lymphoma, not otherwise specified: a report of 340 cases from the International Peripheral T-cell Lymphoma Project. Blood. (2011) 117:3402–8. 10.1182/blood-2010-09-310342 21270441

[73] Iqbal J Wright G Wang C Rosenwald A Gascoyne RD Weisenburger DD. Gene expression signatures delineate biological and prognostic subgroups in peripheral T-cell lymphoma. Blood. (2014) 123:2915–23. 10.1182/blood-2013-11-536359 24632715 PMC4014836

[74] Wang T Feldman AL Wada DA Lu Y Polk A Briski R. GATA-3 expression identifies a high-risk subset of PTCL, NOS with distinct molecular and clinical features. Blood. (2014) 123:3007–15. 10.1182/blood-2013-12-544809 24497534 PMC4014843

[75] Heavican TB Bouska A Yu J Lone W Amador C Gong Q. Genetic drivers of oncogenic pathways in molecular subgroups of peripheral T-cell lymphoma. Blood. (2019) 133:1664–76. 10.1182/blood-2018-09-872549 30782609 PMC6460420

[76] Amador C Greiner TC Heavican TB Smith LM Galvis KT Lone W. Reproducing the molecular subclassification of peripheral T-cell lymphoma-NOS by immunohistochemistry. Blood. (2019) 134:2159–70. 10.1182/blood.2019000779 31562134 PMC6908831

[77] Abate F da Silva-Almeida AC Zairis S Robles-Valero J Couronne L Khiabanian H Quinn SA. Activating mutations and translocations in the guanine exchange factor VAV1 in peripheral T-cell lymphomas. Proc Natl Acad Sci USA. (2017) 114:764–9. 10.1073/pnas.1608839114 28062691 PMC5278460

[78] Abdel-Wahab O Levine RL. Mutations in epigenetic modifiers in the pathogenesis and therapy of acute myeloid leukemia. Blood. (2013) 121:3563–72. 10.1182/blood-2013-01-451781 23640996 PMC3643757

[79] Iqbal J Weisenburger DD Greiner TC Vose JM McKeithan T Kucuk C. Molecular signatures to improve diagnosis in peripheral T-cell lymphoma and prognostication in angioimmunoblastic T-cell lymphoma. Blood. (2010) 115:1026–36. 10.1182/blood-2009-06-227579 19965671 PMC2817630

[80] Dierks C Adrian F Fisch P Ma H Maurer H Herchenbach D. The ITK-SYK fusion oncogene induces a T-cell lymphoproliferative disease in mice mimicking human disease. Cancer Res. (2010) 70:6193–204. 10.1158/0008-5472.CAN-08-3719 20670954

[81] Attygalle AD Feldman AL Dogan A. ITK/SYK translocation in angioimmunoblastic T-cell lymphoma. Am J Surg Pathol. (2013) 37:1456–7. 10.1097/PAS.0b013e3182991415 24076779

[82] Schatz JH Horwitz SM Teruya-Feldstein J Lunning MA Viale A Huberman K. Targeted mutational profiling of peripheral T-cell lymphoma not otherwise specified highlights new mechanisms in a heterogeneous pathogenesis. Leukemia. (2015) 29:237–41. 10.1038/leu.2014.261 25257991 PMC4286477

[83] Xu B Liu P. No survival improvement for patients with angioimmunoblastic T-cell lymphoma over the past two decades: a population-based study of 1207 cases. PLoS ONE. (2014) 9:e92585. 10.1371/journal.pone.0092585 24651162 PMC3961418

[84] Ishitsuka K Utsunomiya A Katsuya H Takeuchi S Takatsuka Y Hidaka M. A phase II study of bortezomib in patients with relapsed or refractory aggressive adult T-cell leukemia/lymphoma. Cancer Sci. (2015) 106:1219–23. 10.1111/cas.12735 26179770 PMC4582992

[85] Feldman AL Sun DX Law ME Novak AJ Attygalle AD Thorland EC. Overexpression of Syk tyrosine kinase in peripheral T-cell lymphomas. Leukemia. (2008) 22:1139–43. 10.1038/leu.2008.77 18401419 PMC2778211

[86] Wilcox RA Sun DX Novak A Dogan A Ansell SM Feldman AL. Inhibition of Syk protein tyrosine kinase induces apoptosis and blocks proliferation in T-cell non-Hodgkin's lymphoma cell lines. Leukemia. (2010) 24:229–32. 10.1038/leu.2009.198 19776763 PMC3122132

[87] Mosse YP Voss SD Lim MS Rolland D Minard CG Fox E. Targeting ALK with crizotinib in pediatric anaplastic large cell lymphoma and inflammatory myofibroblastic tumor: a children's oncology group study. J Clin Oncol. (2017) 35:3215–21. 10.1200/JCO.2017.73.4830 28787259 PMC5617123

[88] Laimer D Dolznig H Kollmann K Vesely PW Schlederer M Merkel O. PDGFR blockade is a rational and effective therapy for NPM-ALK–driven lymphomas. Nat Med. (2012) 18:1699–704. 10.1038/nm.2966 23064464

