# Supplementary material for: Genomics of Peripheral T-Cell Lymphoma and Its Implications for Personalized Medicine
Source: Front Oncol. 2020 Jun 19;10:898. doi: 10.3389/fonc.2020.00898 (PMC7317006; doi:10.3389/fonc.2020.00898)
Supplement: Supplementary file 1 [file Table_1.docx]

Supplementary Material

# Supplementary Figures and Tables

| **Supplementary Table 1. Summary of previous genetic studies in the 3 most common types of PTCL** | | |
| --- | --- | --- |
| **Citation** | **Techniques used** | **Patient Sample Size** |
| (9) | **TDS** | **85** |
| (12) | TDS | 50 |
| (11) | TDS | 92 |
| (17) | WGS and WES, then TDS | 85 |
| (20) | WTS; then TDS | 105 |
| (23) | WES and WTS; then SS | 45 |
| (30) | TDS | 130 |
| (39) | WES, WGS | 11 |
| (43) | WGS, WES; then SS, RNAS | 155 |
| (44) | WES, then TDS | 229 |
| (46) | RNAS | 170 |
| (47) | TDS | 64 |
| (54) | TDS | 190 |
| (56) | WES, TDS, and SS | 170 |
| (55) | WES, then RNAS, TDS | 79 |
| (57) | TDS | 98 |
| (59) | qRT-PCR | 38 |
| (65) | WES, TDS, RNAS, qRT-PCR, and GEP | 133 |
| (66) | qRT-PCR | 73 |
| **Abbreviations:** GEP, gene expression profiling, qRT-PCR, real time quantitative polymerase chain reaction; RNAS, RNA sequencing; SS, Sanger Sequencing, TDS, targeted deep sequencing; WES, whole exome sequencing; WGS, whole genome sequencing; WTS, whole transcriptome sequencing. | | |
